# MicroRNA-26a-mediated regulation of interleukin-2 expression in transformed avian lymphocyte lines

**DOI:** 10.1186/1475-2867-10-15

**Published:** 2010-05-04

**Authors:** Hongtao Xu, Yongxiu Yao, Lorraine P Smith, Venugopal Nair

**Affiliations:** 1 Avian Oncogenic Virus Group, Avian Infectious Diseases Programme, Institute for Animal Health, Compton, Berkshire, UK RG20 7NN

## Abstract

**Background:**

Micro(mi)RNAs are a class of small non-coding RNAs that play critical roles in the induction of various cancers, including lymphomas induced by oncogenic viruses. While some of the miRNAs are oncogenic, miRNAs such as miR-26a are consistently downregulated in a number of cancers, demonstrating their potential tumor suppressor functions. Global miRNA expression profiles of a number of virus-transformed avian lymphoma cell lines have shown downregulation of gga-miR-26a expression, irrespective of molecular mechanisms of transformation or the viral aetiology. The neoplastic transformation of lymphocytes by many viruses accompanies high levels of proliferative responses, mostly mediated through cytokines such as IL-2. Chicken IL-2 can modulate T-cell proliferation and cytotoxicity *in vitro *and *in vivo *and dysregulation of IL-2 expression is observed in diseases such as leukaemia.

**Results:**

The expression levels of gga-miR-26a in chicken lymphoma cells transformed by 3 distinct avian oncogenic viruses, *viz *Marek's disease virus (MDV), avian leukosis virus (ALV) and Reticuloendotheliosis virus (REV) were consistently downregulated compared to the levels in the normal lymphocytes. This downregulation of miR-26a regardless of the viral etiology and molecular mechanisms of transformation was consistent with the tumor suppressor role of this miRNA. Notwithstanding this well-established role in cancer, we demonstrate the additional role of this miRNA in directly targeting chicken IL-2 through reporter and biochemical assays. The downregulation of miR-26a can relieve the suppressive effect of this miRNA on IL-2 expression.

**Conclusions:**

We show that miR-26a is globally downregulated in a number of avian lymphoma cells irrespective of the mechanisms of transformation, reiterating the highly conserved tumor suppressor function of this miRNA. However, with the potential for directly targeting chicken IL-2, the downregulation of miR-26a in these tumor cells could relieve the inhibitory effect on IL-2 expression assisting in the proliferative features of the transformed lymphocyte lines.

## Background

Micro(mi)RNAs are a large class of ~22-nucleotide non-coding RNA molecules that participate a major part in the regulation of gene expression in majority of the eukaryotes. Increasingly, they have been shown to play significant roles in a variety of cancers, in particular of those involving different blood cells [1]. Marek's disease (MD), a naturally occurring CD4+ T-cell lymphoma in chickens induced by Marek's disease virus (MDV), is considered to be a very good model for herpesvirus-induced rapid-onset T-cell lymphomas [2]. Recent studies on the miRNA expression profiles of a number of MDV-transformed chicken lymphoid cell lines have shown significant alterations in the expression of several host miRNAs compared to the normal chicken lymphocytes [3]. One of the miRNAs that was consistently downregulated in a number of MDV-transformed chicken lymphoid cell lines is gga-miR-26a [3]. Suppression of miR-26a has been demonstrated in a variety of human cancers also [4-6] suggesting that miR-26a has potential tumour suppression functions, and its downregulation could be essential for transformation. This notion is supported from the roles of miR-26a in p53 tumour suppressor network [7], as well as in the regulation of transformation-related targets such as cyclin D2, SMAD1, EZH2 and PTEN [8,9].

Antigen-specific T cell proliferation and immunological responses are dependent on the expression of several cytokine genes such as interleukin-2 (IL-2). IL-2 plays an important role in the development, differentiation and homeostasis of T cells, and IL-2 expression is dysregulated in diseases such as leukaemia, autoimmunity and pathogenesis of viral diseases [10], including MD [11]. As in mammals, the chicken IL-2 can modulate T-cell proliferation and cytotoxicity *in vitro *and *in vivo *[12]. The mechanisms of transcriptional regulation of IL-2 promoters through activating transcription factors such as NFκB or AP-1 have been extensively studied [13]. Negative regulation of IL-2 expression is also important both for maintaining the gene in an inactive state in resting cells and for repressing the gene after the activation. Compared to the studies on transactivation, the mechanisms involved in the negative regulation of IL-2 gene expression are less well studied. Although the roles of co-repressors and histone deacetylases in the transcriptional repression of IL-2 has been demonstrated [14], it is also becoming clear that a number of miRNAs are also involved in shaping of the immune responses [15,16] at least in part through the regulation of cytokine genes [17]. For example, miR-146a has been shown to modulate the adaptive immune responses by regulating the IL-2 expression in human T lymphocytes [18]. However, chicken IL-2 is not a predicted target of gga-miR-146a http://www.ebi.ac.uk/enright-srv/microcosm/htdocs/targets/. Although miR-26a has not been implicated in the regulation of IL-2, we examined whether miR-26a downregulation in MDV-transformed chicken lymphoma cell lines do affect IL-2 expression. We present the data suggesting that chicken IL-2 is a direct target of miR-26a, and its downregulation could affect IL-2 expression and signalling pathways in these transformed cells.

## Results

### Transformed chicken cell lines show downregulation of miR-26a

**Figure 1:**
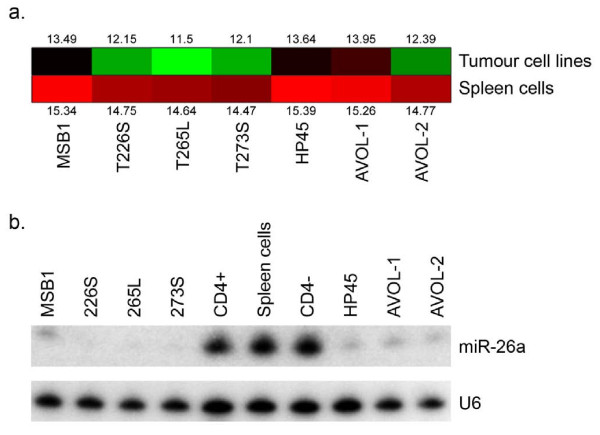
**Downregulation of gga-miR-26a in transformed cell lines**. Reduced expression of miR-26a in MDV (MSB-1, T226S, T265L and T273S), ALV (HP45) and REV (AVOL1 and AVOL2)-transformed cell lines. (a) Heat map of miR-26a expression in tumour cell lines compared with the levels in splenocytes as the reference. Log_2 _values of increased (red) and reduced (green) expression are shown. (b) Northern blot analysis of total RNA extracted from the above cell lines and unsorted splenocytes or sorted CD4^+^/CD4^- ^cells with the anti-miR-26a probe showing differences in the miR-26a expression. U6 RNA was used as the control probe for loading control.

Comparative miRNA expression profiles of 7 MDV-transformed T-cell lines showed that host-encoded miRNAs such as miR-26a, miR-223, miR-150, miR-451 and miR-126 were consistently downregulated [3]. We have now extended the analysis to examine the levels of miR-26a in four of the above MDV-transformed cell lines together with ALV-transformed cell line HP45 and REV-transformed cell lines AVOL1 and AVOL2. The microarray readouts of miRNA expression confirmed that the miR-26a levels in all of these cell lines were lower than the levels in normal splenocytes, although the levels of reduction varied between cell lines (Fig. 1a). Further validation of the reduced expression of miR-26a in these transformed cells was demonstrated by Northern blotting analysis on RNA extracted from these cell lines together with normal CD4+, CD4- and unsorted splenocytes (Fig 1b). The levels of miR-26a signal detected were very high in normal splenocytes, with no major differences between CD4+ and CD4- T cell populations. In contrast, the signals of miR-26a were very low in all transformed cell lines, including those transformed by ALV and REV. In some MD lymphoma lines (T226S, T265L & T273S), the miR-26a levels were undetectable.

### Targeting of chicken IL-2 by miR-26a in reporter assays

**Figure 2:**
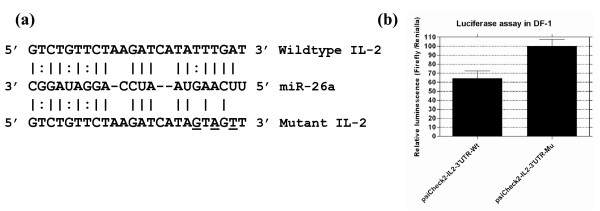
**Chicken IL-2 is a target for miR-26a**. (a) Nucleotide sequences of the wild-type and mutant miR-26a binding sites (mutated residues underlined) located in the 3' UTR of *Gallus gallus *IL-2 gene (NM_204153). (b) Reporter assays on DF-1 cells transfected with the reporter vectors containing either the wildtype or mutated IL-2 3' UTR. Reduction in the ratio of Renilla to Firefly luciferase levels (p < 0.05) in the wildtype construct to the normalised 100 per cent levels for the mutant construct is shown.

To gain insights into the biological functions of miR-26a, we carried out bioinformatic analysis to identify potential targets of miR-26a by scanning the chicken 3' UTR sequences using the miRanda http://www.microRNA.org for potential targets of miR-26a. This analysis predicted that the 3' UTR of chicken IL-2 contained a binding site for miR-26a showing its potential as miR-26a target (Fig. 2a). For further validation of targeting of chicken IL-2 by miR-26a, we constructed reporter vectors with wildtype (wt) or a mutant 3' UTR sequence of the chicken IL-2 with three base-pair mutations in the predicted miRNA binding seed region (Mu) fused to the Renilla luciferase gene in psiCHECK-2 vector (Fig. 2a). Reporter assays on DF-1 cells that express endogenous miR-26a demonstrated that the luciferase reporter levels in the cells transfected with the Wt-3' UTR reporter construct was reduced by nearly 35% (p < 0.05) compared to the Mu-3' UTR construct (Fig. 2b).

### Downregulation of IL-2 expression by miR-26a

**Figure 3:**
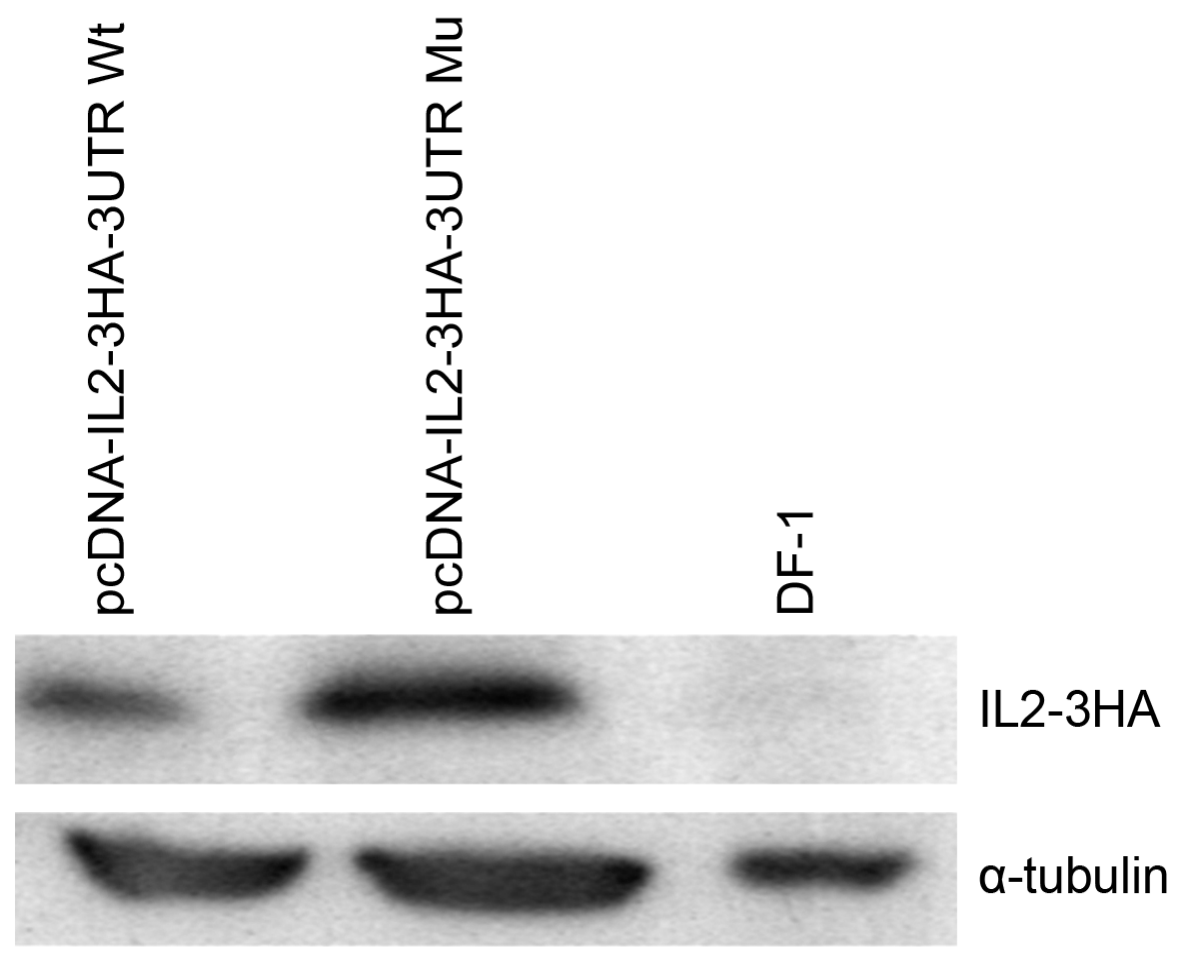
**Western blot analysis of the IL-2 expression**. DF-1 cells transfected with pcDNA-IL-2-3HA-3'UTR wild-type or mutant constructs were detected using anti-HA tag antibody. The levels of α-tubulin in the cell lysates are also shown as the loading control.

As we did not have an antibody that specifically detected the chicken IL-2, we constructed vectors that expressed HA tagged-chicken IL-2 with either the wildtype 3' UTR sequence or with miR-26a binding site mutant sequence. Western blot analysis of the IL-2 expression levels with anti-HA antibody in DF-1 cells transfected with the Wt or the mu IL-2 expression constructs showed that the IL-2 levels in constructs expressing the Wt 3' UTR was reduced by more than 20% compared to the Mu constructs (Fig 3), showing a direct silencing of IL-2 expression by miR-26a.

### Levels of miR-26a affect IL-2 expression

**Figure 4:**
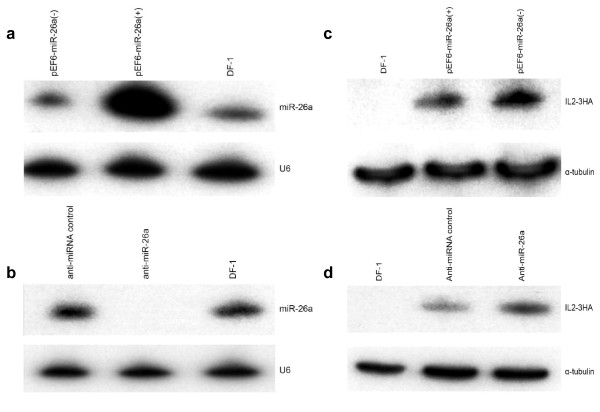
**Regulation of chicken IL-2 expression by miR-26a in DF-1 cells**. (a) Northern blot analysis of total RNA extracted from DF-1 cells transfected with miR-26a expression or control vectors using anti-miR-26a probe. (b) Northern blot analysis demonstrating the specific silencing of miR-26a expression with anti-miR-26a, but not with anti-miRNA control. U6 RNA was used as the control probe for loading control. (c) Western blot analysis of chicken IL-2 expression in DF-1 cells co-transfected with HA-tagged IL-2 expression construct pcDNA-IL-2-3HA-3'UTR and miR-26a expression vector (lane 2) or control vector (lane 3) using anti-HA tag antibody. (d) Western blot analysis of the IL-2 levels in the presence of anti-miR-26a or anti-miRNA control with anti-HA tag antibody. The levels of α-tubulin in the cell lysates are also shown as the loading control. Quantitation of the band densities was carried out using ImageQuant 300 software.

For further evidence to show that IL-2 levels are regulated by miR-26a, we performed experiment in which the miR-26a levels in DF-1 cells are either upregulated by overexpression or downregulated by specific silencing. For overexpression, we transfected DF-1 cells with the miR-26a expression construct pEF6-miR-26a (+). As the negative control, we used the construct pEF6-miR-26a (-), in which the miRNA was cloned in the reverse orientation. Northern blot analysis of DF-1 cells transfected with the two vectors showed that the miR-26a expression level of pEF6-miR-26a (+) transfected cells was higher than that of normal DF-1 cells or the cells transfected with the pEF6-miR-26a (-) vector (Fig. 4a). For downregulation of miR-26a, anti-miR-26a construct was transfected into DF-1 cells. Northern blot showed that anti-miR-26a dramatically reduced miR-26a levels compared to negative control (Fig. 4b).

We also examined how the changes in the expression of miR-26a affected the IL-2 expression. MiR-26a expression vector and control vector were respectively co-transfected with pcDNA-IL-2-3HA-3'UTR into DF-1, and then cells were examined by western blot analysis (Fig. 4c). It showed that the IL-2 expression level was reduced by more than 30% in the presence of miR-26a compared to the levels in cells transfected with pEF6-miR-26a (-) control vector. In contrast, the IL-2 expression level was increased by more than 140% when the miR-26a levels were silenced using the anti-miR-26a compared to the anti-miRNA control (Fig.4d).

## Discussion

Over the last 5 years, miRNAs have emerged as major regulators of gene expression and cancer pathogenesis. Nearly all of the tumours, irrespective of the cell type or etiology, have shown globally abnormal miRNA expression patterns and miRNA profiles of tumours have given valuable insights into downstream pathways of oncogenesis [19-25]. We have previously demonstrated that miR-26a is downregulated in seven independently-derived MDV-transformed lymphoblastoid T cell lines [3]. Here, we show that such downregulation of miR-26a occur in lymphoid cell lines transformed by ALV and REV also, suggesting that miR-26a-mediated modulation of gene expression is probably crucial for lymphocyte transformation, irrespective of the viral etiology. The identification of some of the validated targets of miR-26a including EZH2 [9], SMAD1 [26], PTEN [8] provides intriguing link to the oncogenic pathways. Loss of expression of miR-26a also occurs in some of the human tumours such as thyroid anaplastic carcinoma [6] and Burkitt's lymphoma [9] reiterating the role of miR-26a as a tumour suppressor, the expression of which can protect from the disease progression in cancer models [5,27].

In this study we demonstrate that, in addition to its potential role as a tumour suppressor, miR-26a also functions as a modulator of IL-2 expression. IL-2 is produced mainly by activated T helper CD4+ lymphocytes and is one of critical cytokines that control the proliferation and clonal expansion of transformed lymphoma cells [28]. In MDV-transformed tumour cells, MDV-encoded oncoprotein Meq is thought to play a direct role in the upregulation of IL-2 expression by binding to the IL-2 promoter [29]. Our study shows that the downregulation of miR-26a is another global pathway for the increased IL-2 expression in the lymphoma cell lines transformed by the avian oncogenic viruses MDV, ALV and REV.

Our study does not examine the molecular mechanisms of downregulation of miR-26a. Some of the previous studies have shown the role of oncogenes such as c-*myc *in regulating miR-26a expression [4,30,31] and the altered expression of miR-26a in HP45 could be related to the increased c-*myc *expression. Similarly, the reduced expression of miR-26a in AVOL1 and AVOL2 cell lines may also be mediated by the v-*rel*. In MD tumour cell lines, the MDV-encoded oncoprotein MEQ is thought to be the primary determinant of transformation [32]. Presence of a putative MEQ-binding sequence ACACA at -650 nucleotide position would suggest a role of MEQ in modulating the miR-26a expression, although further studies are required validate this.

## Conclusions

In summary, we show that miR-26a is globally downregulated in a number of avian lymphoma cells transformed by different oncogenic viruses that use multiple pathways for inducing transformation. In addition to its role as a global tumor suppressor miRNA, we also provide data to show that chicken IL-2 is a direct target for miR-26a. Our study suggests that the suppression of miR-26a could potentially relieve the inhibitory effect on IL-2 expression and could contribute to the proliferative features of the transformed lymphocyte lines.

## Methods

### Analysis of miRNA expression

Details of the microarray experiments carried out using small RNA samples prepared from transformed lymphoblastoid cell lines, normal splenocytes were labelled with either Cy3 or Cy5 197 dyes using the Array 900microRNA RT kit from Genisphere (Hatfield, PA) and hybridized to μRNA microarray have been described [33]. For Northern blot analysis, 20 μg of total RNA extracted from cultured cells by using TRIzol reagent (Invitrogen) were resolved on a 15% polyacrylamide urea gel and blotted onto a GeneScreen Plus membrane (Perkin-Elmer). DNA oligonucleotide with the complementary sequence to miR-26a was end labelled with [γ-^32^P] ATP by T4 polynucleotide kinase (New England Biolabs). Hybridization and autoradiography were carried out using standard methods as previously described [34].

### Construction of reporter vectors

The region containing the IL-2 3'UTR, amplified from chicken spleen genomic DNA by PCR using the forward primer (5'-CTCGAGGCAACTAATCATTTTTATTTTAC TGC-3') and the reverse primer (5'-CATATATTACTGAAATTTATTAAATG-3'), was cloned initially into pGEM-T Easy vector (Promega). The IL-2 3' UTR mutant with three nucleotides altered in seed sequence was constructed using the Quikchange™ site-directed mutagenesis kit (Stratagene CA) using the forward primer (5'-CTGCATGGACCTAACATTCGATGATCATTCAGTTTAATAGG TTAAACTGCAATTGA-3') and reverse primer (5' TCAATTGCAGTTTAACCTAT TAAACTGAATGATCATCGAATGTTAGGTCCATGCAG-3'). After confirming the sequence of the wild type and mutant regions of the IL-2 3'UTR, these were cloned downstream of the Renilla luciferase gene through the *Not*I - *Xho*I site in psiCheck-2 vector (Promega) to generate psiCheck2-IL-2 3' UTR-Wt and psiCheck2-IL-2 3' UTR-Mu reporter constructs.

### Construction of miR-26a expression vectors

For the construction of gga-miR-26a expression plasmids, the miR-26a primary gene together with ~200-bp flanking sequences were amplified from chicken genomic DNA using primer pairs (5'-ATGTTCTTTAATGTCGGGAGC-3') and (5'-AAAGAATTCTGCCCGTGAC-3'), and cloned into pEF6-V5/His TOPO vector (Invitrogen) under control of EF1α promoter vector. Another construct where the sequence was cloned in the reverse orientation was used as the negative control.

### Construction of chicken IL-2 expression vectors

As we did not have the specific antibodies that detect the chicken IL-2, we constructed IL-2 expression vector with a C-terminal HA tag and the 3'UTR. For this, the IL-2 gene, amplified by PCR with the forward primer (5'-AAGCTTGCCACCAT GATGTGCAAAGTACTGATC-3') that included the Kozak sequence and *Hind*III site and reverse primer (5'-CTCGAGTTAAGCGTAATCTGGAACATCGTAT GGGTATGCCATTTTTTGCAGATATCTCAC-3') carrying the haemagglutinin (HA) tag sequence and *Xho*I site, was initially cloned into pGEM-T Easy vector. The IL-2 gene and 3'UTR regions were ligated by *Xho*I and *Not*I and cloned into *Hind*III- *Not*I site in pcDNA3.1 vector to construct the IL-2 expression vector with the C-terminal HA tag and the 3' UTR.

### Cell culture, reporter assays and miRNA expression

MDV-transformed lymphoid cell lines MSB-1, 226S, 265L and 273S, avian leukosis virus (ALV)-transformed cell line HP45, and reticuloendotheliosis virus (REV)-transformed cell lines AVOL1 and AVOL2 have been described [3]. Chicken embryo fibroblast cell line DF-1 [35] was used for reporter assays and expression studies. The transfection of DF-1 cells was carried out with Lipofectamine 2000 (Invitrogen) as per manufacturer's protocols. Approximately 5 × 10^4 ^DF-1 cells were seeded in each well of a 96-well plate. Purified DNA (100 nanograms) of the reporter vectors were transfected into DF-1 cells in triplicates and luciferase assay was performed using the Dual Glo Luciferase Assay System (Promega) 24-hour after transfection. The relative expression levels were calculated as a ratio of the target specific Renilla luciferase to the levels the Firefly luciferase. For the expression of miR-26a, DF-1 cells (0.5 × 10^6 ^in each well) of 6-well plate were transfected with 2 μg of the expression constructs. For silencing miR-26a expression, 5 nM of anti-gga-miR-26a or Cy-3-labelled control anti-miRNA were transfected into DF-1, and analysed by Northern blot 48 hours later.

### Analysis of IL-2 expression

Approximately 2 μg each of the purified DNA of the IL-2 expression vectors were transfected into each well of DF-1 cells in a 6-well plate and cell lysates collected 48 hours later for western blot analysis. For functional analysis of miR-26a-mediated silencing, IL-2 (500 ng) and miR-26a (2 μg) expression vectors were co-transfected into DF-1 cells. In addition, DF-1 cells co-transfected with 5 nM each of anti-gga-miR-26a or Cy-3 labelled control anti-miRNA (Ambion) with miR-26a expression or control vectors were also used in these studies.

For western blot analysis, cells lysates in protein gel sample buffer (8 M urea, 2% sodium dodecyl sulfate, 10 mM Tris-HCl [pH 6.8], 0.05% bromophenol blue) and separated on a NuPAGE 4-12% bis-Tris gel (Invitrogen) and transferred onto nitrocellulose membranes using an iBlot gel transfer system (Invitrogen). Specific detection of IL-2 was carried out using mouse HA.11 monoclonal antibody (Covance) and rabbit anti-mouse IgG peroxidase conjugate (Dako). The alpha-tubulin levels detected with the specific monoclonal antibody was used as the loading control. Membranes were developed with an ECL Western blotting system (Amersham) and images quantified using ImageQuant 300 software (GE Healthcare).

## Competing interests

The authors declare that they have no competing interests.

## Authors' contributions

**HX **carried out most of the experiments, **YY **provided the microarray data and Northern blot analysis, **LPS **carried out all the tissue culture work on cell lines and **VN **secured the funding, designed the experiments and contributed to the writing of the manuscript

All authors have read and approved the final manuscript.
